# High-resolution greenspace dynamic data cube from Sentinel-2 satellites over 1028 global major cities

**DOI:** 10.1038/s41597-024-03746-7

**Published:** 2024-08-22

**Authors:** Shengbiao Wu, Yimeng Song, Jiafu An, Chen Lin, Bin Chen

**Affiliations:** 1 Future Urbanity & Sustainable Environment (FUSE) Lab, Division of Landscape Architecture, Department of Architecture, Faculty of Architecture, The University of Hong Kong, Pok Fu Lam, Hong Kong SAR, China; 2 School of the Environment, Yale University, New Haven, CT 06511 USA; 3 Department of Real Estate and Construction, The University of Hong Kong, Pok Fu Lam, Hong Kong SAR, China; 4 Institute for Climate and Carbon Neutrality, The University of Hong Kong, Pok Fu Lam, Hong Kong SAR, China; 5 Faculty of Business and Economics, The University of Hong Kong, Pok Fu Lam, Hong Kong SAR, China; 6 Urban Systems Institute, The University of Hong Kong, Pok Fu Lam, Hong Kong SAR, China

**Keywords:** Sustainability, Forestry

## Abstract

Greenspace, offering multifaceted ecological and socioeconomic benefits to the nature system and human society, is integral to the 11^th^ Sustainable Development Goal pertaining to cities and communities. Spatially and temporally explicit information on greenspace is a premise to gauge the balance between its supply and demand. However, existing efforts on urban greenspace mapping primarily focus on specific time points or baseline years without well considering seasonal fluctuations, which obscures our knowledge of greenspace’s spatiotemporal dynamics in urban settings. Here, we combined spectral unmixing approach, time-series phenology modeling, and Sentinel-2 satellite images with a 10-m resolution and nearly 5-day revisit cycle to generate a four-year (2019–2022) 10-m and 10-day resolution greenspace dynamic data cube over 1028 global major cities (with an urbanized area >100 km^2^). This data cube can effectively capture greenspace seasonal dynamics across greenspace types, cities, and climate zones. It also can reflect the spatiotemporal dynamics of the cooling effect of greenspace with Landsat land surface temperature data. The developed data cube provides informative data support to investigate the spatiotemporal interactions between greenspace and human society.

## Background & Summary

Greenspace, broadly defined as a land surface covered with trees, shrubs, grass, and any other vegetation types, is one of the key environmental components in urban areas and provides a wide range of ecological and socioeconomic benefits^1,2^. For example, the existence of greenspace in cities offers vital ecosystem services through a variety of pathways, such as improving air quality^3,4^, reducing noise pollution and flooding risks^5–8^, mitigating thermal stress from heatwaves and extreme weather events^9,10^, and promoting urban biodiversity conservation in forms of revegetation^11^. Specific greenspace types, such as old or giant trees, are reserved for religious or cultural traditions^12^. At the same time, being exposed to greenspace has widely proven to be beneficial to residents’ physical and mental health^13^ via promoting physical activities^14,15^, increasing recreational entertainment^16,17^, reducing stress^18,19^, and stimulating societal cohesion^20,21^. Given these recognized greenspace benefits, the United Nations has specified the need of providing universal access to greenspace for urban residents in the 11^th^ Sustainable Development Goal of Cities and Communities^22^.

Greenspace supply, as a proxy of measuring the spatial distribution, abundance, composition, and configuration of greenspace, has been widely applied in urban studies and practices to understand greenspace settings and their interactions with human society. A growing body of studies have been conducted to capture greenspace supply and demand by measuring greenspace availability, accessibility, and visibility to humans^23–26^. With the greenspace-supply framework, the critical environmental injustice in terms of greenspace exposure inequality across multiple scales has been persistently reported^27,28^. In local contexts, access to greenspace is disproportionately lower for communities characterized by lower income, less education, and higher percentage of people of color^29–32^. This inequality in greenspace supply will be translated into disparities in human health such as thermal stress mitigation benefits^33,34^ and mental well-being^19,35^. On a broader scale, there is a significant contrast in greenspace exposure between cities in Global South and North. Global South cities have only a third of the greenspace exposure level that Global North cities enjoy, yet experience almost double the inequality level of the latter^36–38^. These instances of environmental injustices underscore the crucial role of socioeconomic factors in urban greening design and management, beyond just biophysical considerations for city planners and landscape architects. Therefore, on a critical role, the effectiveness of measures to enhance greenspace supply requires the provision of spatially and temporally explicit information about existing urban greenspace.

Geographic information systems (GIS) and remote sensing technology have greatly facilitated the mapping and monitoring of greenspace at local, city, regional, and even global scales. Street-view imaging provides local details on the greenspace environment from an eye-level perspective by using identification and classification approaches^39,40^. However, due to discrete sampling of viewing geometry, this approach only captures a small spatial extent of greenspace without completely explicit information^41,42^. Satellite imagery, on the other hand, substantially expands our capability of mapping and monitoring greenspace from a local eye-view to a bird-view perspective at regional and global scopes. Two major approaches have been developed for satellite-based greenspace mapping, including the hard classification method that attributes each pixel into a green or non-green type with the supervised or unsupervised strategy^43,44^, and the soft fractional estimate method that extracts sub-pixel greenspace coverage with the spectral unmixing or machine learning approaches^45–47^. The hard classification approach is often used with the land cover classification product with greenspace-specific information such as the National Land Cover Database^48,49^. Owing to the capability of capturing greenspace features beyond pixel resolution, the soft fractional estimate approach turns to be more promising for large-scale satellite-based urban greenspace mapping^24,27,47^.

Despite many advancements in satellite-based greenspace mapping, urban greenspace phenology, as a natural cycle, remains not fully accounted for or understood. Greenspace is dynamic with an annual recurring rhythm of several key stages, including leaf emergence, green-up, flowering, leaf senescence, and leaf-off^50^. Latitudinal gradients of greenspace phenology controlled by temperature and other biotic or abiotic disturbances are widely recognized^51^. Such phenology difference inevitably leads to seasonal variations in the balance between greenspace supply and demand across cities. Consider the temperate city of Beijing (39°55′N, 116°23′E) and the subtropical city of Shenzhen (22°33′N, 114° 03′E) as examples, assuming identical levels of greenspace coverage and peak volume, residents in Beijing would experience bare winter branches, while those in Shenzhen would have access to year-round greenspace^23^. Beyond the amount of greenspace (i.e., quantity), phenology also influences the quality of greenspace through changes in leaf color^52^. For example, spring bloom and autumn colors are more visually aesthetic and attractive to the public than the uniform green of summer^53^. A key challenge for urban greenspace phenology mapping, particularly when employing the soft fractional estimate approach, is the lack of high-resolution time-series phenology dataset. The daily Moderate Resolution Imaging Spectroradiometer (MODIS) satellite, with 500-m spatial resolution, cannot adequately monitor urban greenspace^54^. Moreover, the recent emerging 3-m resolution daily commercial PlanetScope satellite faces limitations due to large data volume^55^. Given this context, this study will develop a phenology-informed framework to generate a high-resolution greenspace dynamic data cube for fine-scale urban greenspace monitoring.

## Methods

### Global cities

**Fig. 1:**
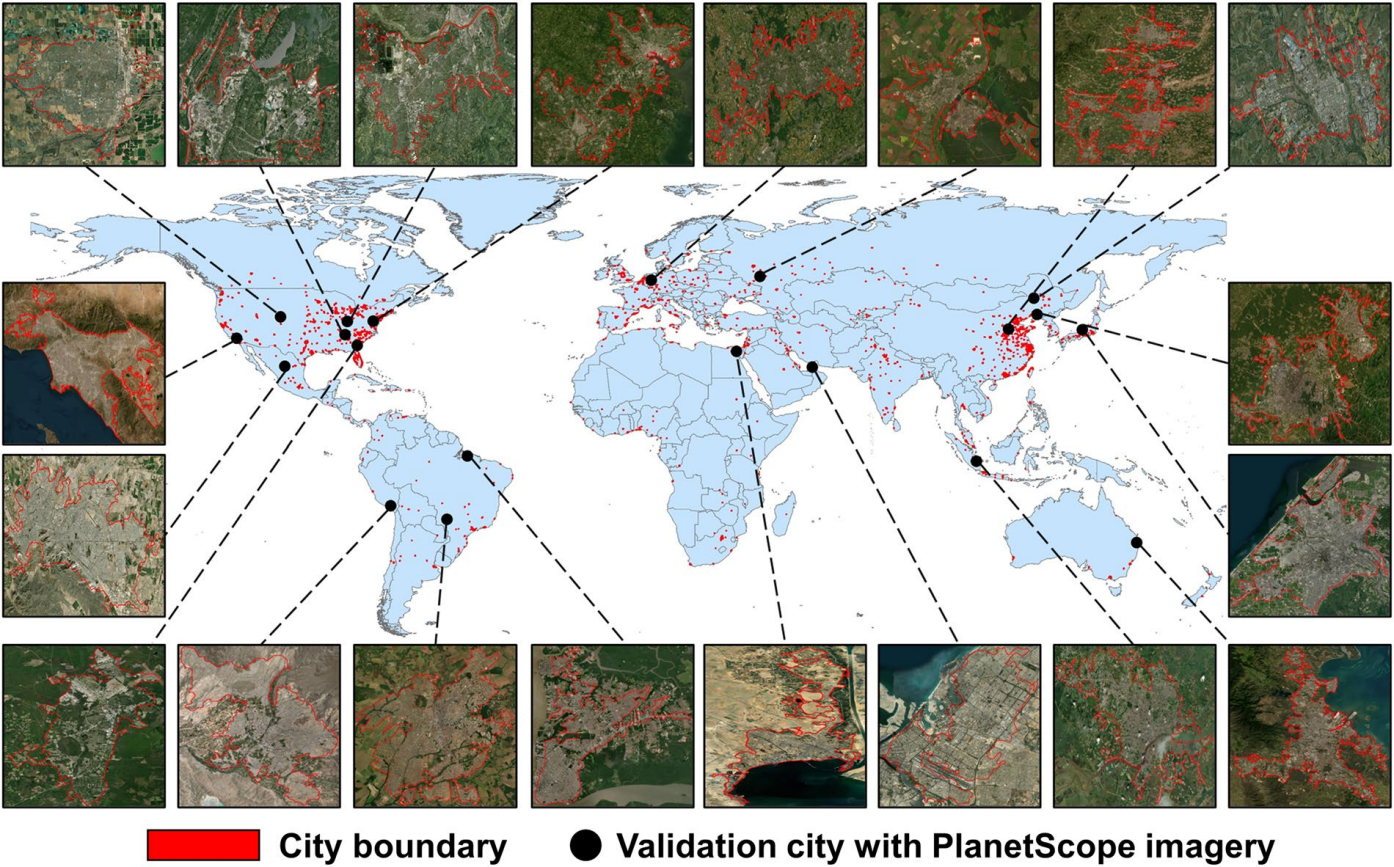
Geographic distribution of 1028 global cities (red polygons) used for Sentinel-2-based spatiotemporal greenspace data cube mapping and 20 validation cities (black dots) with PlanetScope surface reflectance data. The city boundaries are extracted from the latest Global Urban Boundaries vector dataset for 2018^56^. Administrative boundaries are extracted from the Global Administrative Areas (GADM) data (https://gadm.org/). The RGB true-color images of validation cities are extracted from the ESRI World Imagery Basemap (https://www.esri.com/en-us/home).

This study focused on 1028 major urban areas worldwide (i.e., an urbanized area >100 km^2^), which were identified and extracted from the latest Global Urban Boundaries vector dataset for 2018^56^. These urban areas are distributed between the developed and developing regions, with 522 in Global North and 506 in Global South. These city samples were also geographically diverse in different climate zones, with the highest number of urban areas (530) in subtropic, followed by temperate (329), and tropic (169) areas. Despite using physical built-up areas instead of administrative boundaries to extract our urban areas, we referred to them as “cities” throughout the manuscript for the city-level analysis (Fig. 1).

### Satellite datasets

We used Sentinel-2 imagery from 2019 to 2022 to generate time series of greenspace dynamics (referred to as spatiotemporal greenspace data cube afterward) over 1028 global major cities. The Sentinel-2 satellites collect surface reflectance data over the globe between 56°S–84°N, with a spatial resolution ranging from 10 to 60 meters and a revisit cycle of around 5 days^57^. Specifically, the 10-m resolution data includes one near-infrared and three visible (blue, green, and red) spectral bands. Before 2019, Sentinel-2 satellite surface reflectance was mainly available for North American and European regions. Therefore, we used the Sentinel-2 satellite data from 2019 onwards. The Google Earth Engine (GEE) platform provides Sentinel-2 surface reflectance data at https://developers.google.com/earth-engine/datasets/catalog/COPERNICUS_S2_SR^58^ and the associated cloud probability product at https://developers.google.com/earth-engine/tutorials/community/sentinel-2-s2cloudless.

We used the 3-m resolution PlanetScope satellite images in 2020 (Fig. 1) for the accuracy validation of Sentinel-2-derived spatiotemporal greenspace data cube. PlanetScope satellite is operated by the commercial Planet Labs Inc. with daily frequency and 3-m spatial resolution^55^. To enable worldwide assessment, we selected 20 cities as validation sites using a random sample strategy according to the city density and ensuring their global coverage. We downloaded PlanetScope imagery for each city in two steps. We first filtered high-quality PlanetScope imagery with cloud coverage lower than 5% and sensor-viewing geometry smaller than 10° for each month. Based on these data, we selected a one-day observation per month that has an overlapping area fraction with the validation city boundary of over 90%. To match the Sentinel-2 spectral bands, we used four-band (blue, green, red, and near-infrared) PlanetScope surface reflectance data and calculated the NDVI metric as greenspace reference, which relative bias is reported to be less than 6.2%^59^. Additionally, we used the fractional greenspace of Sentinel-2 data over global 1028 cities for 2020 as an additional reference. Specifically, we filtered out Sentienl-2 data over each city for the second 10^th^ day interval (e.g., DOY 11–20 for January; DOY 41–50 for February etc.) within each month and conducted linear spectral unmixing analysis on them to extract the mean fractional greenspace as benchmark. We then compared the Sentinel-2-derived fractional greenspace with our greenspace data cube for pixel-level accuracy assessment.

### Framework of Sentinel-2-derived spatiotemporal greenspace data cube

**Fig. 2:**
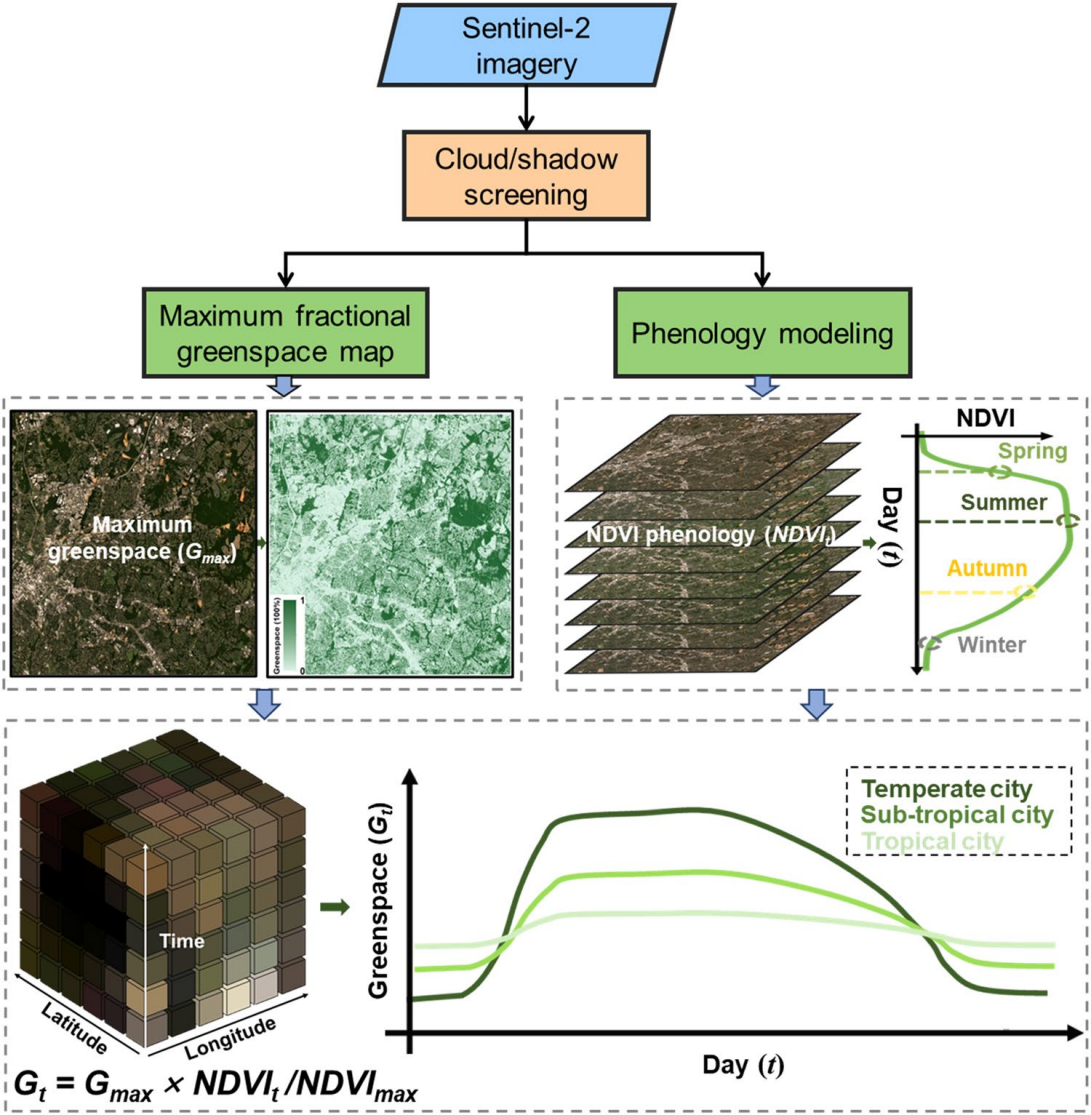
Flowchart for generating spatiotemporal greenspace data cube from Sentinel-2 satellite.

Our algorithm flowchart includes two major parts: annual maximum estimation of greenspace coverage and phenology modeling of NDVI time series (Fig. 2). NDVI and sub-pixel fractional greenspace derived from the linear spectral unmixing approach are two widely used metrics to quantify greenspace coverage^27,60^. We here broadly defined greenspace as the sub-pixel fractional greenspace percentage because of its fine details for small green patches at the sub-pixel level, and assumed greenspace coverage seasonality can be measured by the NDVI phenology (see Supplementary Material). With this assumption, we modeled the spatiotemporal dynamics of greenspace data cube by combing the annual maximum greenspace coverage and NDVI phenology as follows:

1 $${G}_{t}={G}_{\max }\times \frac{{{NDVI}}_{t}}{{{NDVI}}_{\max }}$$

where *G*_*t*_ is the greenspace coverage at day *t* expressed as the day-of-year (DOY), *NDVI*_*t*_ represents the NDVI value at day *t*, *G*_*max*_ and *NDVI*_*max*_ are the annual maximum extents of greenspace coverage and NDVI, respectively.

### Annual maximum estimation of greenspace coverage

We extracted the maximum extent of greenspace coverage from the annual composite of cloud-free Sentinel-2 satellite time series using the linear spectral unmixing approach^27,36^. First, the cloud-free Sentinel-2 imagery is generated from the raw data by cloud masking with the pixel-level cloud probability product. We then created a six-band time-series feature image by adding the NDVI and normalized difference water index (NDWI) as two additional features to the four-band surface spectral reflectance. Based on the six-band time-series feature map, we generated the greenness composite on a pixel-by-pixel basis using the NDVI feature as an ordering metric. To detect the greenspace coverage that includes small green patches (e.g., street trees), we adopted a linear spectral unmixing approach to extract sub-pixel fractional greenspace from the greenest composite of Sentinel-2 data as the maximum greenspace coverage.

This linear spectral unmixing model assumes that the spectral feature of each Sentinel-2 pixel can be modeled as a linear combination of a group of spectrally pure endmembers^46^.

2 $${S}_{i}=\mathop{\sum }\limits_{k=1}^{n}{f}_{ik}\,\cdot \,{C}_{ik}+{\varepsilon }_{i}$$

where *S*_*i*_ represents the six spectral signatures of pixel *i*, *C*_*ik*_ represents the spectral signature of the *k*th endmember in the *i*th pixel, *ε*_*i*_ is the unmodeled residual in the *i*th pixel, *n* is the total number of endmembers, *f*_*ik*_ is the fraction of *k*th endmember within pixel *i*, which is usually calculated from the least-squares method with the following physical constraints:

3 $$\mathop{\sum }\limits_{k=1}^{n}{f}_{{ik}}=1\,{and}\,{f}_{{ik}}\ge 0\,\forall k=1,\cdots ,n$$

Here, we selected three spectral endmembers in Eq. (3) (i.e., *n* = 3): vegetation, bare surface, and water body, to drive the linear spectral unmixing process.

To account for the cross-year spectral feature variations for each endmember, we included three empirical constraints: (i) vegetation endmember with an NDVI value larger than 0.8^37^, (ii) bare surface endmember with an NDVI value less than 0.2^24^, and (iii) water body with an NDWI value larger than^61^. Based on these constraints, we mapped the candidate regions of three endmembers from the greenest composited images and then identified the final endmember pixels by drawing regions of interest (ROIs) from these potential regions. With the selected endmember pixels, we extracted their spectral features for each year, and then extracted the annual maximum greenspace extent by solving the linear spectral unmixing model in Eq. (2), using the “unmix” algorithm in the Google Earth Engine cloud-computing platform (https://developers.google.com/earth-engine/apidocs/ee-image-unmix).

### Phenology modeling of NDVI time series

We modeled the seasonal phenology of NDVI from Sentinel-2 data with three steps: (i) Based on the NDVI metric time series in Section 2.3.2, we interpolated the data gaps arising from cloud contaminations using the linear approach with the nearest valid observations^62^. (ii) we composited the gap-filled NDVI time series into a 10-day interval by averaging the NDVI value within the time window. (iii) We further used the RMMEH compound smoother^63^ to adjust the outliers of the Sentinel-2 NDVI time series. Consequently, we derived the 10-day smoothed Sentinel-2 NDVI time series. With this NDVI phenology dataset and the annual maximum greenspace coverage, we generated a 10-m spatial, and 10-day temporal resolution spatiotemporal greenspace data cube based on Eq. (1).

## Data Records

Two major datasets are freely available in the figshare repository (https://figshare.com/projects/Greenspace_Seasonality_Data_Cube/190971)^64^, which include:

1. Greenspace data: four-year (2019-2022) 10-m, 10-day resolution greenspace rater data across global 1028 major cities are stored in the GEOTIFF format. Each GEOTIFF file includes 36 spectral bands, representing the number of 10-day intervals within one year. The scaling factor for each band is 0.001, with the physical greenspace coverage ranging from 0 to 1.
2. City boundary: the continuous built-up areas for the 1028 cities are stored in the shapefile vector format, with the metadata information of longitude, latitude, city ID, area, and continent.

## Technical Validation

### Validation of spatiotemporal greenspace data cube

**Fig. 3:**
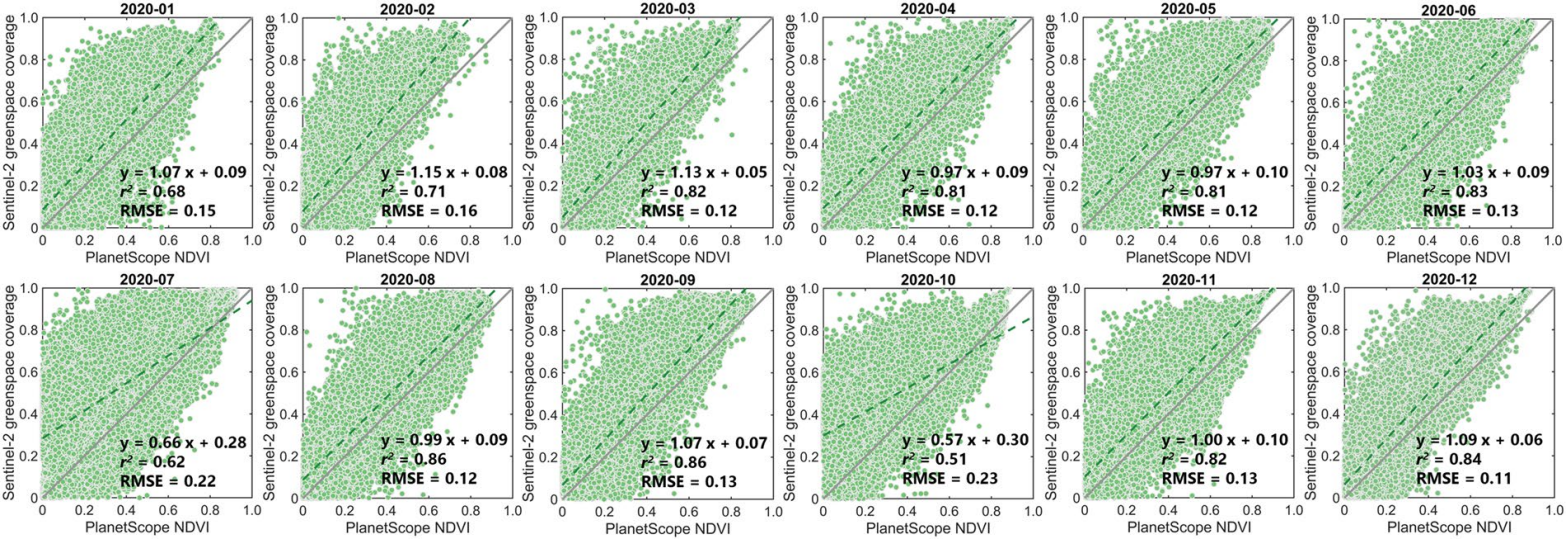
Validation of Sentinel-2 greenspace data cube using PlanetScope fraction greenspace in 2020 as reference. Each green dot represents the 200-m-resolution block-level mean of greenspace coverage.

**Fig. 4:**
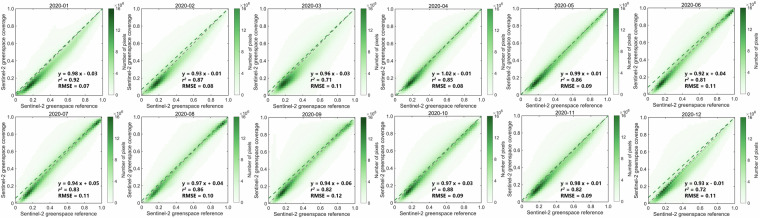
Validation of the Sentinel-2 greenspace data cube using Sentinel-2 fractional greenspace over 12 months in 2020 as reference. Each green dot represents the density results of pixel-level comparison. The greenspace of the second 10^th^ day interval for each month was compared and assessed.

To validate the accuracy of Sentinel-2-derived spatiotemporal greenspace data cube, we used the 3-m resolution NDVI map from PlanetScope over 20 worldwide cities as the greenspace coverage reference because we assume that PlanetScope data can capture the small greenspace patches within Sentienl-2 pixels. Considering the difference between Sentinel-2 and PlanetScope resolution and larger urban area, we divided the spatial coverage of each validation city into a group of 200 m × 200 m blocks as the basic units for greenspace comparison, following the previous protocol^36^. We extracted the block-level PlanetScope NDVI means and compared them with the corresponding month Sentinel-2 greenspace coverage. The coefficient of determination metric (i.e., *r*^2^) was used to measure the greenspace coverage accuracy. With the fraction greenspace reference derived from PlanetScope, the Sentinel-2-derived greenspace shows a comparable accuracy (Fig. 3), with the RMSE ranging from 0.11–0.23 and *r*^2^ ranging from 0.51–0.86, despite some uncertainties existing due to the inconsistent time between PlanetScope reference and Sentinel-2 greenspace data cube. The assessment results with reference from Sentinel-2 fractional greenspace also confirmed high accuracy of the proposed Sentinel-2 greenspace data cube, with the RMSE ranging from 0.07-0.12 and *r*^2^ ranging from 0.71–0.92 (Fig. 4).

**Fig. 5:**
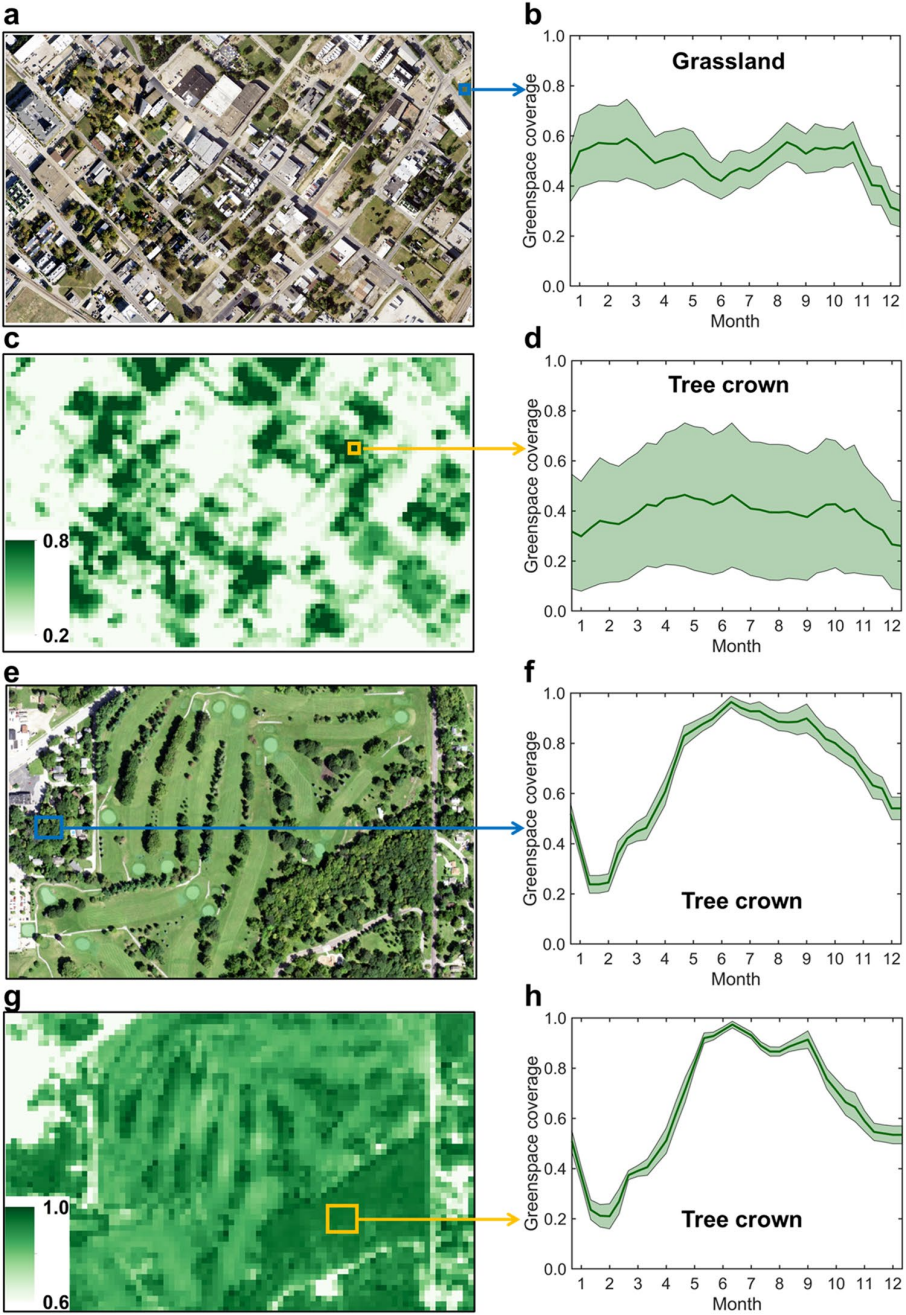
Visual assessment of the capability of Sentinel-2-derived spatiotemporal greenspace data cube to monitor fine-scale spatiotemporal dynamics of greenspace in Dallas (**a**–**d**) and Des Moines (**e**–**h**), with (**a,e**) 1-m resolution National Agriculture Imagery Program (NAIP) imagery on June 20, 2020 and August 22, 2019, (**b,d,f,h**) greenspace seasonality, and (**c,g**) Sentinel-2-derived greenspace on DOY 170-180. Shading denotes the standard deviation of greenspace change.

We also downloaded the 1-m resolution National Agriculture Imagery Program (NAIP) imagery for Dallas (32°46′45″N 96°48′32″W), North Texas, United States on June 20, 2020, and Des Moines (41°35′12″N, 93°37′30″W), Iowa, United States on August 22, 2019, as the reference for visualization assessment (Fig. 5). The visual inspection reveals the spatial distribution of Sentinel-2 greenspace is very consistent with that of NAIP imagery (Fig. 5a,c,e,g). Moreover, the proposed Sentinel-2 spatiotemporal greenspace data cube can monitor fine-scale greenspace seasonality, such as tree crown phenology with a single growth peak in summer and grassland phenology with two growth peaks in both spring and autumn (Fig. 5b,d,f,g).

### Spatiotemporal analysis of greenspace

**Fig. 6:**
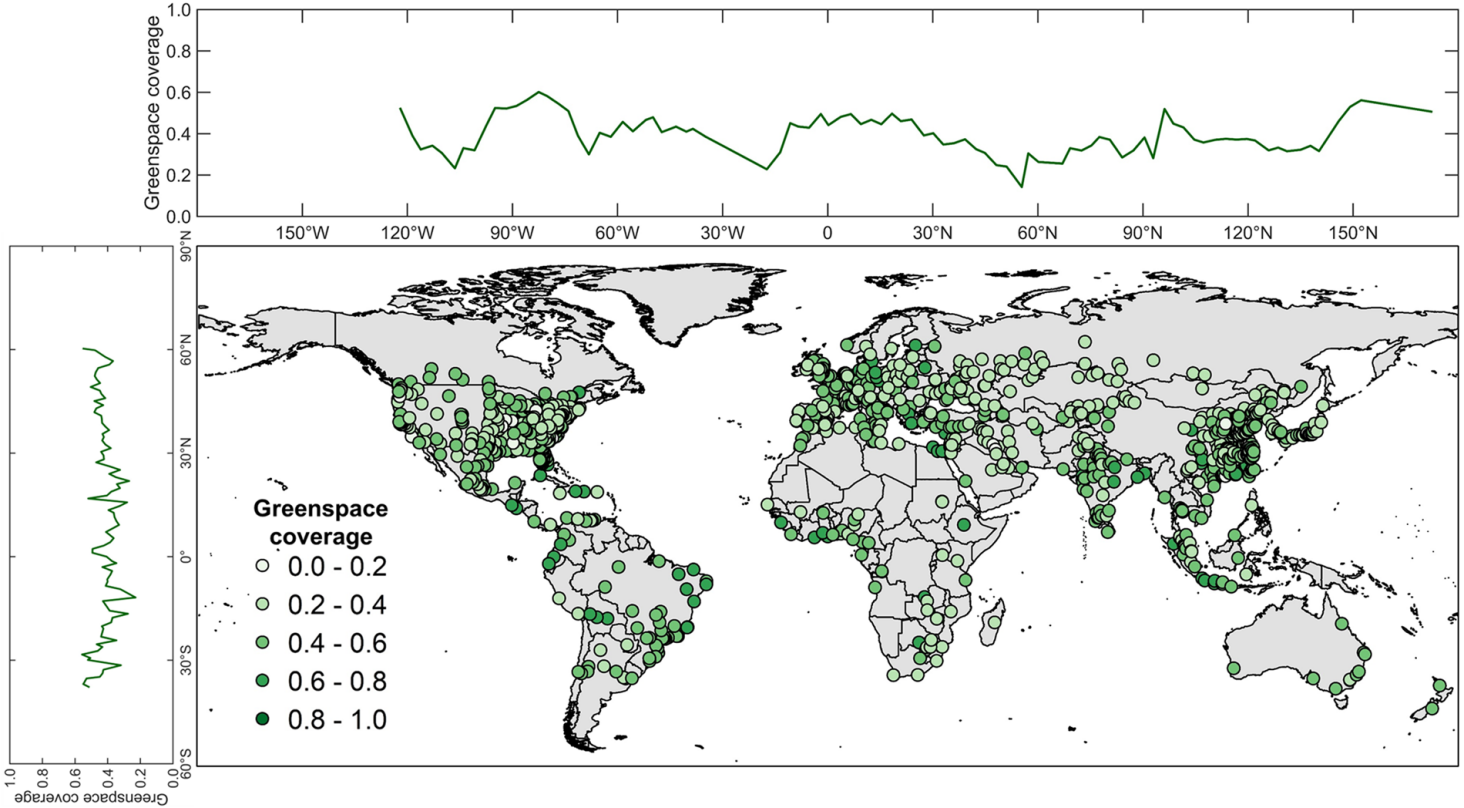
Spatial map of annual mean city-level greenspace in 2020 and the associated longitudinal and latitudinal gradients. The gradient plots are calculated by the bin-averaged with a 1st percentile interval for longitude and latitude regions.

**Fig. 7:**
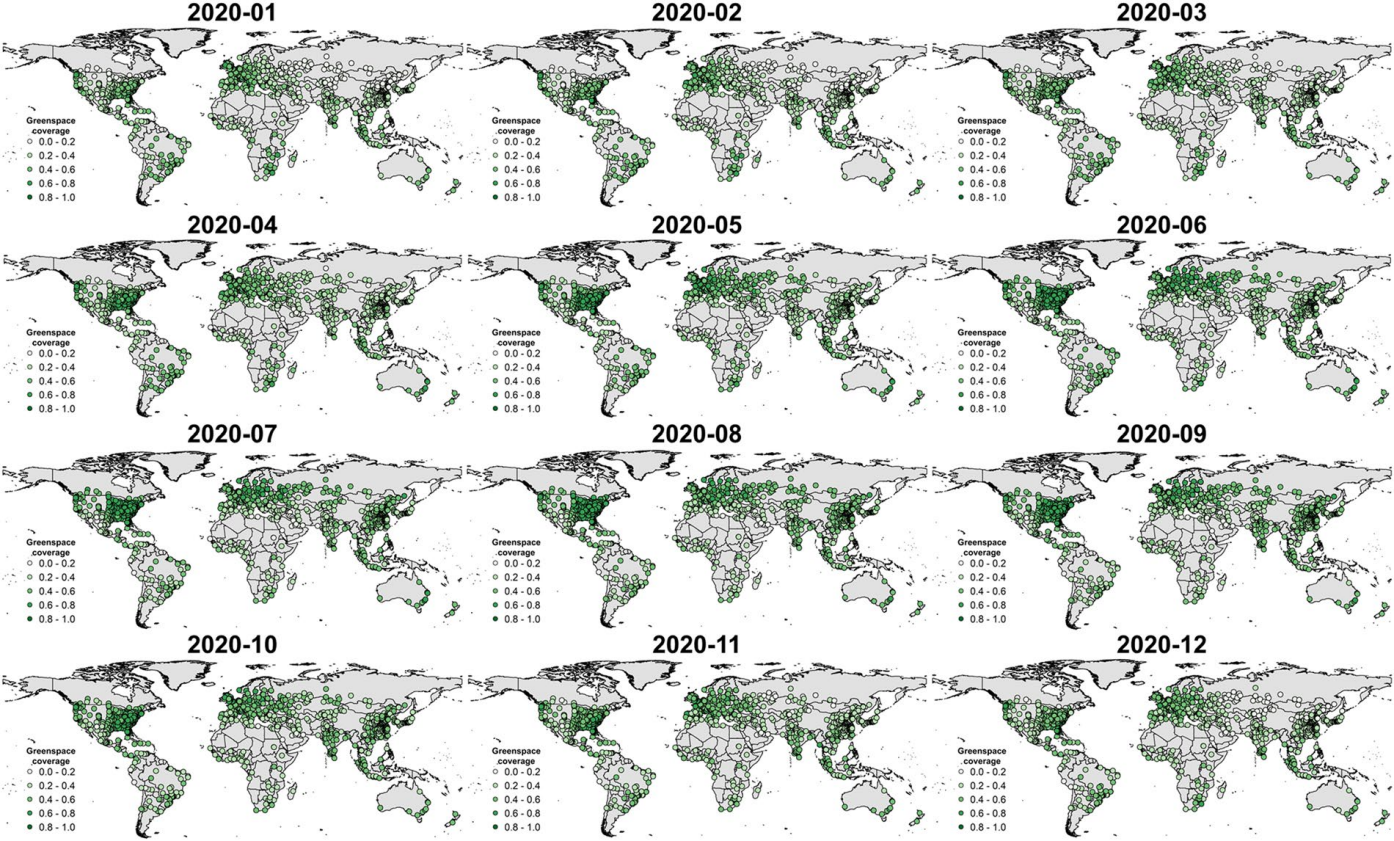
Spatial maps of monthly city-level greenspace in 2020 by aggregating the 10-day Sentinel-2-derived spatiotemporal greenspace data cube to a one-month frequency.

According to the annual mean city-level greenspace coverage in 2020, a total of 953 (92.7%) cities has a mean greenspace coverage level lower than 0.6 (Fig. 6). A spatial aggregation of city-level greenspace coverage can be observed, with higher greenspace coverages in North American, Austrian, European, South American, and Southeast Asian cities. In contrast, Middle Eastern and African cities have relatively lower greenspace coverage. These spatial aggregation patterns are also revealed by the longitudinal and latitudinal gradient curves of greenspace coverage. Besides the spatial heterogeneity, greenspace coverage is highly temporally dynamic (Fig. 7). For example, the cities of high latitude in the North hemisphere show clear greenspace seasonality, with the greenspace coverage lowest in winter, peaking in summer, and medium magnitude levels in spring and autumn. Compared to the high-latitude cities, the equatorial cities are much less affected by seasonality.

**Fig. 8:**
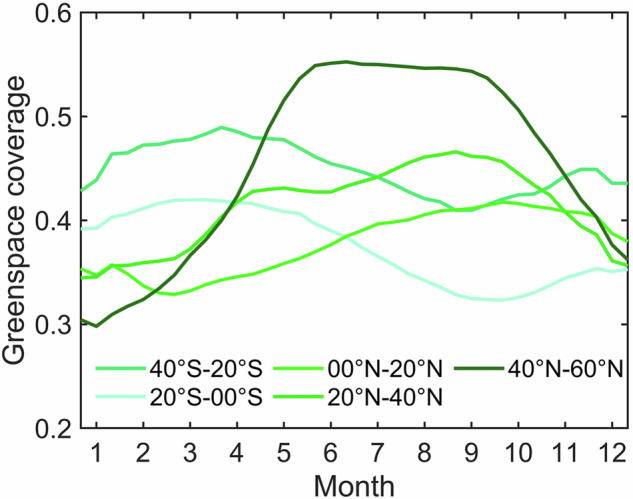
Seasonal dynamic of city-level mean greenspace in 2020 across 5 climate zones, including temperate (40°N-60°N), subtropical (−40°S-20°S and 20°N-40°N), and tropical (−20°S-0°S and 0°N-20°N).

**Fig. 9:**
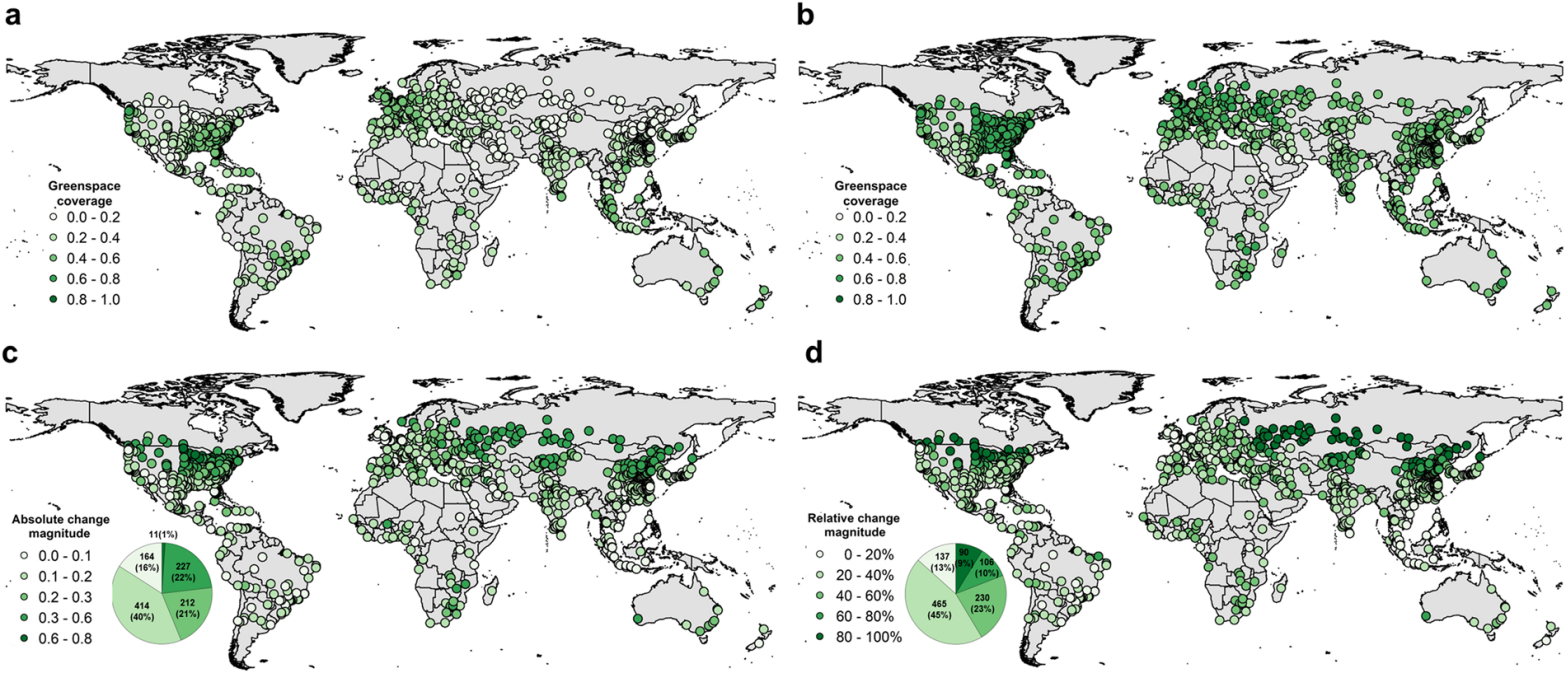
The influence of seasonal phenology on city-level greenspace monitoring in 2020 for (**a**) minimum greenspace coverage, (**b**) maximum greenspace coverage, (**c**) absolute change magnitude of seasonal greenspace coverage, and (**d**) relative change magnitude of seasonal greenspace coverage. In (**c,d**), the pie chart presents the city distribution (number and fraction) for each interval of magnitude change.

The greenspace seasonality patterns across latitude gradients in 2020 are diverse (Fig. 8). Tropical (20°S-0°S and 0°N-20°N) and subtropical (40°S-20°S and 20°N-40°N) cities show similar seasonal patterns, with the greenspace coverage growth peaking in spring while at the nadir in autumn, and tropical cities are greener than subtropical cities. The Northern hemispherical cities have the opposite seasonality patterns from the Southern hemispherical cities but experience a similar magnitude level. The temperate (40°N–60°N) cities are more temporally dynamic than tropical and subtropical cities, with a shorter growing and browning season but a longer peaking stage. To further quantify the seasonal impacts on greenspace coverage estimation, we compared the minimum and maximum composited greenspace coverages, which show different spatial patterns across cities (Fig. 9). In the maximum composition scheme, the inter-city greenspace difference is minor, most of which is smaller than 0.2. This difference might be enlarged to 0.4 in the minimum composition scheme (Fig. 9a,b). The absolute change magnitude of greenspace increases with the city’s latitude, with the maximum level of 0.6 in temperate cities, the smallest level of 0.1 in tropical cities, and a total of 238 (23%) cities larger than 0.3 (Fig. 9c). The relative change magnitude follows a similar spatial pattern to the absolute one, with 106 (10%) and 90 (9%) cities over 60% and 80%, respectively (Fig. 9d). These spatiotemporal dynamics of greenspace are also observed by the Sentinel-2 datasets in the other three years (Figs. S1–6). We also found considerable greenspace variations over the years. For instance, the greenspace coverage magnitude in 2022 is lower than those of the other three years (2019–2021).

### Spatiotemporal analysis of greenspace cooling effect

The high-resolution Sentinel-2-derived spatiotemporal greenspace data cube allows for spatiotemporal monitoring of the cooling effects of greenspace. We executed this implication at Dallas by combing the spatiotemporal greenspace data cube and Landsat-8 land surface temperature (LST) product for 2020. We screened the clear-sky Landsat data with a small cloud cover (<10%) and downloaded LST data for 10 days (i.e., DOY = 7, 39, 151, 199, 215, 231, 263, 279, 343, and 359). We matched Landsat LST data with the nearest greenspace data due to their different temporal resolutions. Based on the matched Landsat LST data and greenspace dataset, we mapped pixel-level cooling efficiency (CE, i.e., the magnitude of LST reduction by 1% increase of greenspace coverage)^65^ using a moving window-based regression approach^66^. This approach assumes all pixels within the window have the same climate background so that the LST reduction for these pixels is majorly attributed to the greenspace increase. The moving window size of 5 × 5 Landsat pixels (~150 m × 150 m) was selected in this study, of which the result compared with those of the other window sizes (7 × 7, 9 × 9, 11 × 11, and 13 × 13 Landsat pixels).

**Fig. 10:**
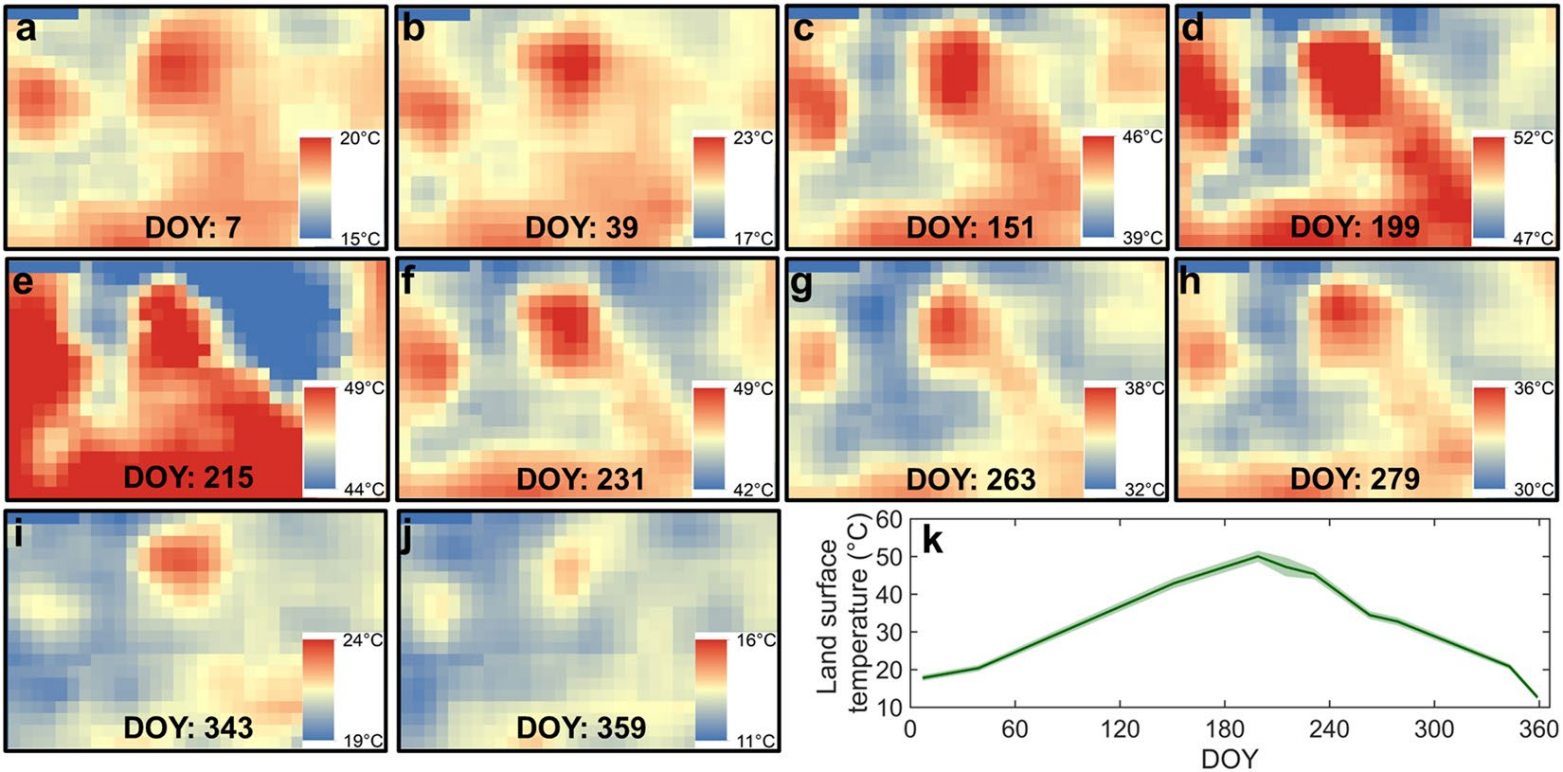
Seasonality of land surface temperature (LST) captured by Landsat-8 satellite in Dallas, with (**a**–**j**) the pixel-level LST map, and (**k**) the seasonality of the mean and standard deviation (shading region) of LST. DOY denotes the day of the year.

**Fig. 11:**
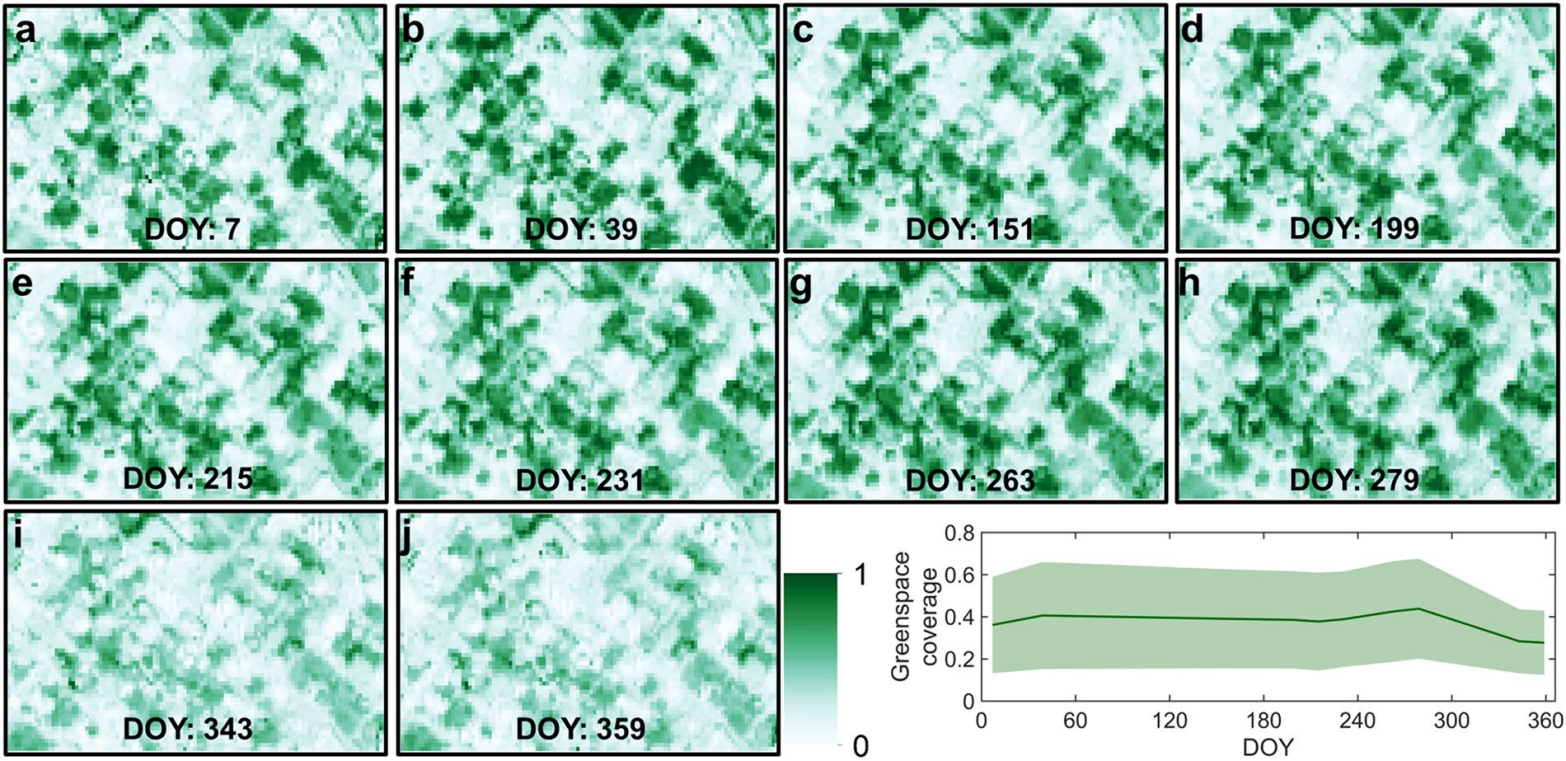
Seasonality of greenspace coverage in Dallas, with (**a**–**j**) the pixel-level greenspace coverage map, and (**k**) the seasonality of the mean and standard deviation (shading region) of greenspace coverage. DOY denotes the day of the year. As the Sentinel-2-derived spatiotemporal greenspace data cube has a 10-day temporal resolution, we selected the greenspace data nearest to LST in Fig. 10 to match the urban thermal dataset.

**Fig. 12:**
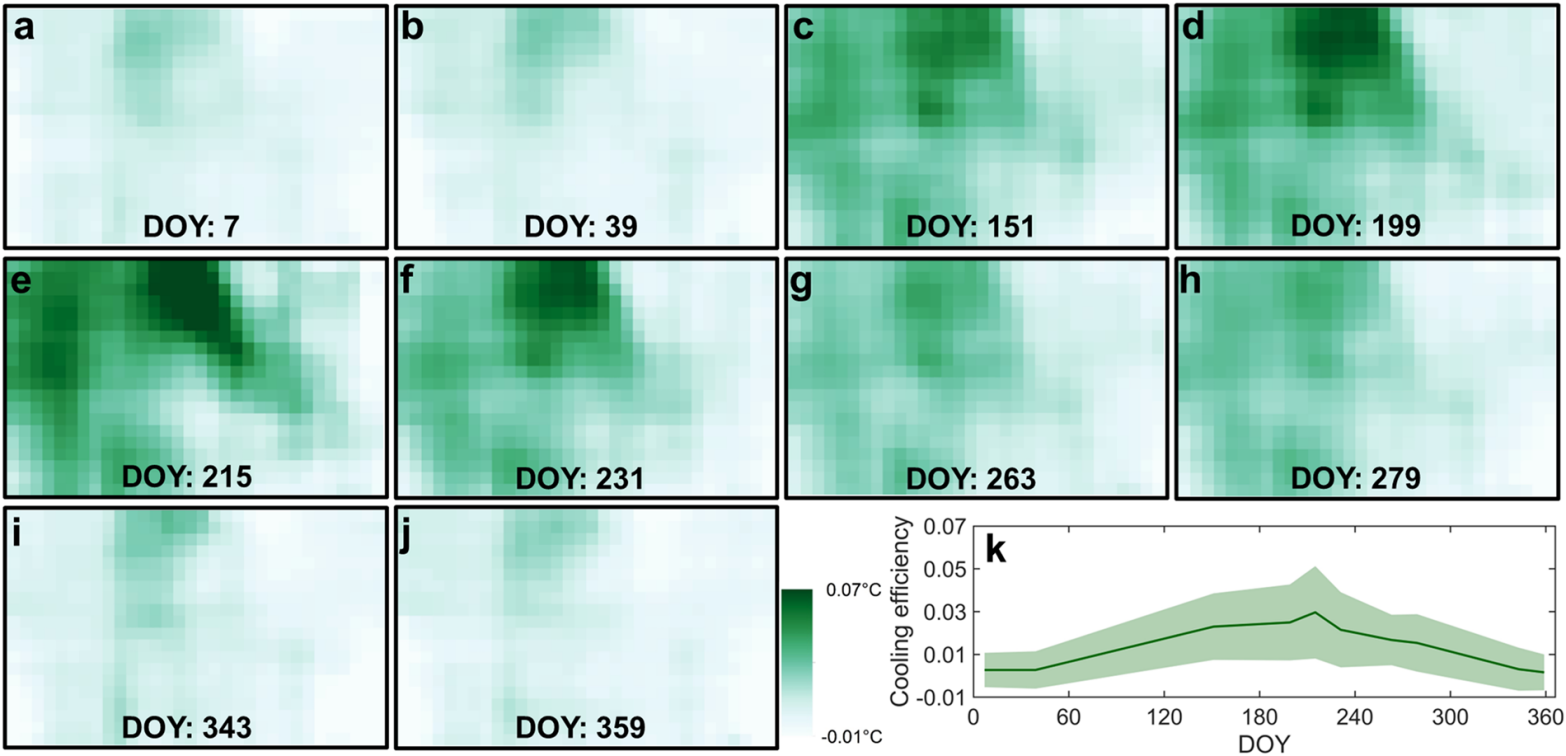
Seasonality of vegetation cooling efficiency (CE) in Dallas, with (**a**–**j**) the pixel-level CE map, and (**k**) the seasonality of the mean and standard deviation (shading region) of CE. The moving window size of 5 × 5 pixels is used for the CE calculation. CE is calculated and shown with the same resolution as the Landsat land surface temperature (LST) data.

Our greenspace data cube can reflect the spatiotemporal heterogeneity of urban thermal environment, as supported by Dallas, a city with a humid subtropical climate (Fig. 10). The impervious surfaces such as buildings and paved roads exhibit much higher temperatures than the green environment such as tree canopies and grasslands (Fig. 10a–j). The city’s mean LST displays a downward V-shape, peaking at about 50.0 °C in the summer and dropping to a minimum of approximately 12.7 °C during the spring and winter (Fig. 10k). Like grassland, the city-level average greenspace coverage shows two local peaks in spring and autumn (Fig. 11). This is attributed to grassland being the dominant greenspace type in this city (Fig. 5a). The CE shows a similar spatial pattern to the LST, with the highest CE values observed in the hottest areas (Fig. 12a–j). Its seasonality also likes that of the LST, peaking at around 0.030 °C in the summer and reaching its lowest at around 0.002 °C in the spring and winter (Fig. 12k). Our sensitivity analysis reveals that the choice of moving window size has minimal impacts on the calculation of greenspace cooling effects (Fig. S7).

## Usage Notes

This pixel-level data cube is suitable for cross-scale greenspace extraction and analysis, and can be used for parcel, neighborhood, and city-scale greenspace management to support urban biodiversity conservation. It provides fine-scale observational data support for the study of greenspace supply-demand dynamics over space and time, and greenspace-based urban cooling that can help urban planners, designers, and policymakers to develop climate adaption strategies for countering local warming and achieving healthy and sustainable living environment.

However, although the high spatial resolution of Sentinel-2 satellite allows fine-scale greenspace monitoring, the quality of Sentinel-2 imagery suffers from some artificial uncertainties of the shadow effects. The shade cast by taller buildings or trees will block the sunshine and sensor viewing of the shorter neighboring vegetated objects and underestimate their reflected signals, resulting in an underestimated even negative NDVI and consequently causing bias in greenspace coverage estimation. The high-resolution Light Detection and Ranging (LiDAR) datasets and photorealistic 3D model can be used to provide more spatially explicit urban shadow map as auxiliary information to reconstruct greenspace information under the shaded regions.

### Supplementary information

SUPPLEMENTARY INFORMATION

## Data Availability

The spatiotemporal greenspace data cube is generated on the Google Earth Engine (GEE) cloud-computing platform. The code for extracting and displaying greenspace coverage is publicly available at: https://code.earthengine.google.com/33b1f787976f25b33fa1146071ea5c3c.
