# Supplementary material for: High-resolution greenspace dynamic data cube from Sentinel-2 satellites over 1028 global major cities
Source: Sci Data. 2024 Aug 22;11:909. doi: 10.1038/s41597-024-03746-7 (PMC11341826; doi:10.1038/s41597-024-03746-7)
Supplement: Supplementary file 1 — SUPPLEMENTARY INFORMATION [file 41597_2024_3746_MOESM1_ESM.docx]

**High-resolution greenspace dynamic data cube from Sentinel-2 satellites over 1028 global major cities**

Shengbiao Wu^1^, Yimeng Song ^2^, Jiafu An^3^, Chen Lin^4^, Bin Chen^1,5,6,*^

1. Future Urbanity & Sustainable Environment (FUSE) Lab, Division of Landscape Architecture, Department of Architecture, Faculty of Architecture, The University of Hong Kong, Hong Kong SAR, China
2. School of the Environment, Yale University, New Haven, CT, 06511, USA
3. Department of Finance and Insurance, Faculty of Business, Lingnan University, Hong Kong SAR, China
4. Faculty of Business and Economics, The University of Hong Kong, Hong Kong SAR, China
5. Urban Systems Institute, The University of Hong Kong, Hong Kong SAR, China
6. Musketeers Foundation Institute of Data Science, The University of Hong Kong, Hong Kong SAR, China

*Corresponding author: Bin Chen ([binley.chen@hku.hk](mailto:binley.chen@hku.hk))

Submission to ***Scientific Data***

**Seasonality modeling of urban greenspace**

We assumed greenspace coverage seasonality can be measured by the NDVI phenology based on the linear spectral unmixing algorithm, which is used for extracting sub-pixel fractional greenspace as shown in the following Eq. (1):

$S_{i}=\sum_{k=1}^{n} f_{ik}\cdot C_{ik}+\varepsilon_{i}$ (1)

where $S_{i}$ represents the six spectral signatures (i.e., blue, green, red, and NIR reflectance, NDVI, and NDWI) of pixel *i*, $C_{ik}$ represents the spectral signature of the *k*th endmember in the *i*th pixel, $\varepsilon_{i}$ is the unmodeled residual in the *i*th pixel, *n* is the total number of endmembers, $f_{ik}$ is the fraction of *k*th endmember within pixel *i,* which is usually calculated from the least-squares method with the following physical constraints:

$\sum_{k=1}^{n} f_{ik}=1 and f_{ik}\geq0 \forall k = 1, \cdots, n$ (2)

where three spectral endmembers (i.e., *n* = 3): vegetation, impervious areas, and water, are used to drive the linear spectral unmixing process.

By expanding Eq. (R1) for NDVI metric at time *t* and *t_max_*, we can obtain Eqs. (3-4) as follows:

${NDVI}_{t}=f_{t,veg}\cdot{NDVI}_{veg}+ f_{t,imper}\cdot{NDVI}_{imper}+ f_{t,water}\cdot{NDVI}_{water}+\varepsilon_{t}$ (3)

${NDVI}_{max}=f_{max,veg}\cdot{NDVI}_{veg}+ f_{max,imper}\cdot{NDVI}_{imper}+ f_{max,water}\cdot{NDVI}_{water}+\varepsilon_{max}$ (4)

Linear spectral unmixing theory implies two physical constraints: 1) distinct spectral signatures of three endmembers, i.e., NDVI_veg_ >> NDVI_imper_ >> NDVI_water_ (e.g., NDVI_veg_ = 0.90, NDVI_imper_ = 0.03, and NDVI_water_ = 0.03 in our data cube extraction for 2020), and 2) trivial unmodeled residual term $\varepsilon$, i.e., $\varepsilon\approx0$. Based on these two constraints, we update Eqs. (3-4) as follows:

${NDVI}_{t}\approx f_{t,veg}\cdot{NDVI}_{veg}=$ $G_{t}\cdot{NDVI}_{veg}$ (5)

${NDVI}_{max}\approx f_{max,veg}\cdot{NDVI}_{veg}=G_{max}\cdot{NDVI}_{veg}$ (6)

Finally, we can approximate the seasonal dynamics of sub-pixel fractional greenspace percentage with NDVI phenology from Eqs. (5-6) as follows:

$G_{t}= G_{max}\times\frac{{NDVI}_{t}}{{NDVI}_{max}}$ (7)


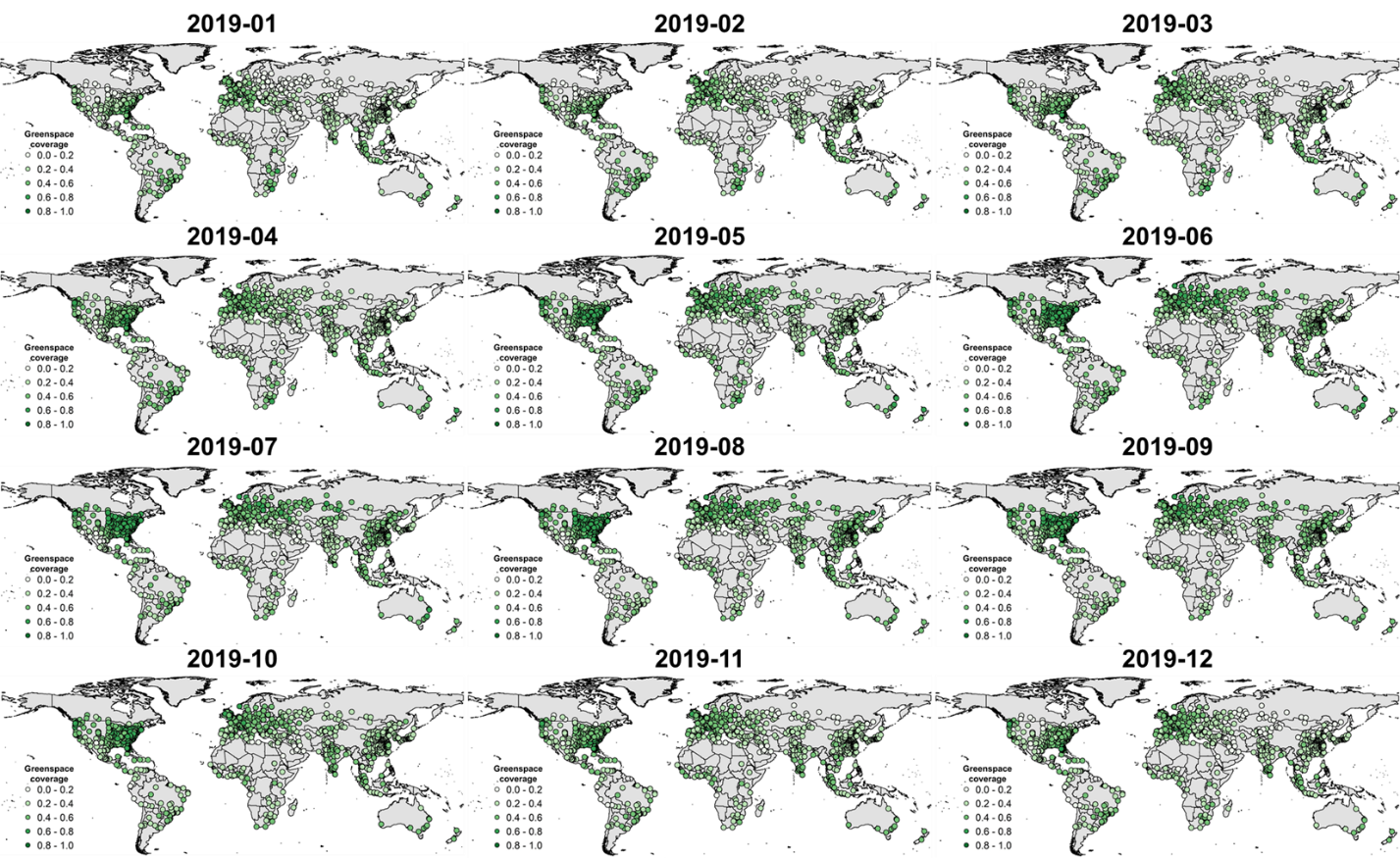


**Fig. S1.** Spatial map of monthly city-level greenspace in 2019 by aggregating the 10-day Sentinel-2 greenspace data cube to a one-month resolution.


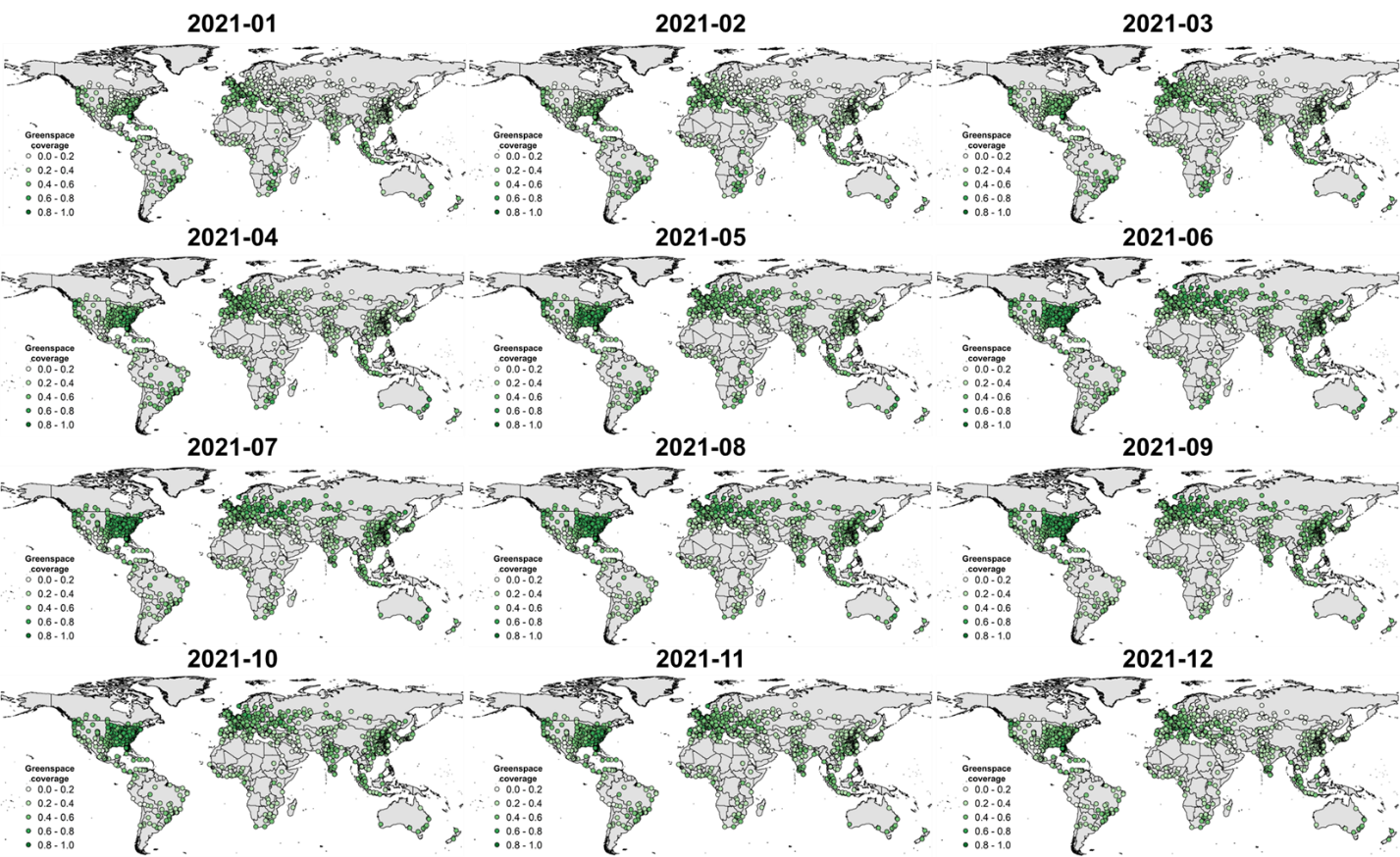


**Fig. S2.** Spatial map of monthly city-level greenspace in 2021 by aggregating the 10-day Sentinel-2 greenspace data cube to a one-month resolution.


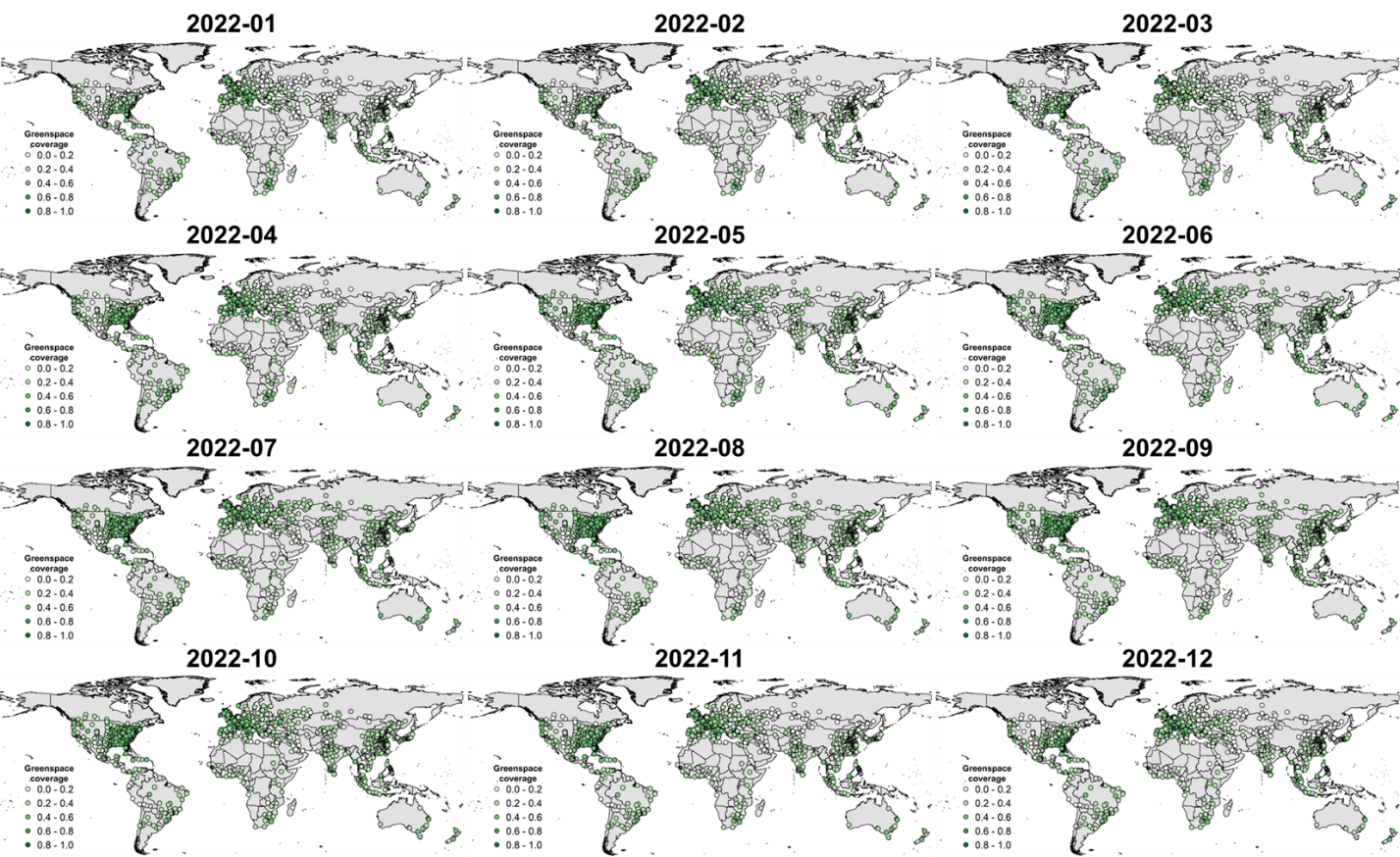


**Fig. S3.** Spatial map of monthly city-level greenspace in 2022 by aggregating the 10-day Sentinel-2 greenspace data cube to a one-month resolution.


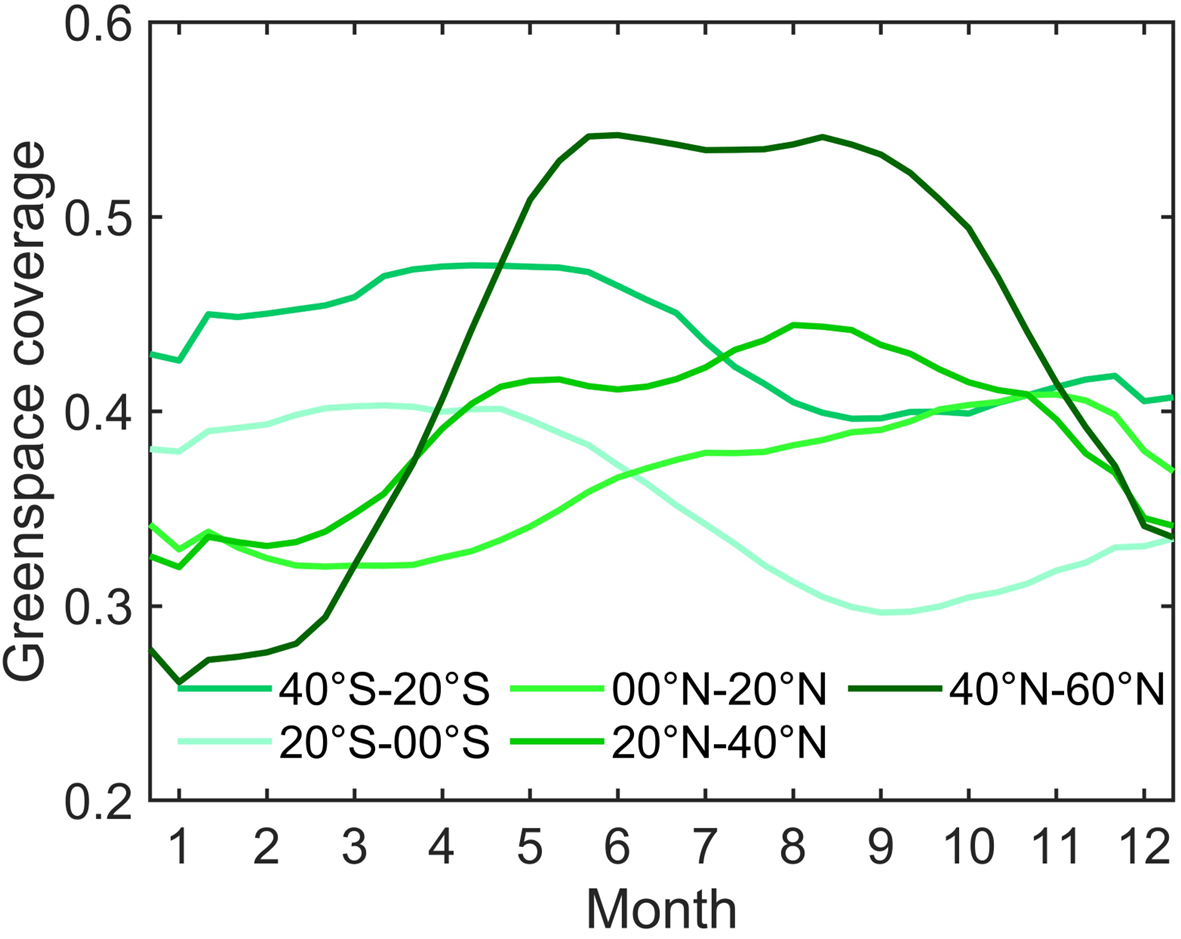


**Fig. S4.** Seasonal dynamic of city-level mean greenspace in 2019 across 5 climate zones, including temperate (40°N-60°N), subtropical (-40°S-20°S and 20°N-40°N), and tropical (-20°S-0°S and 0°N-20°N).


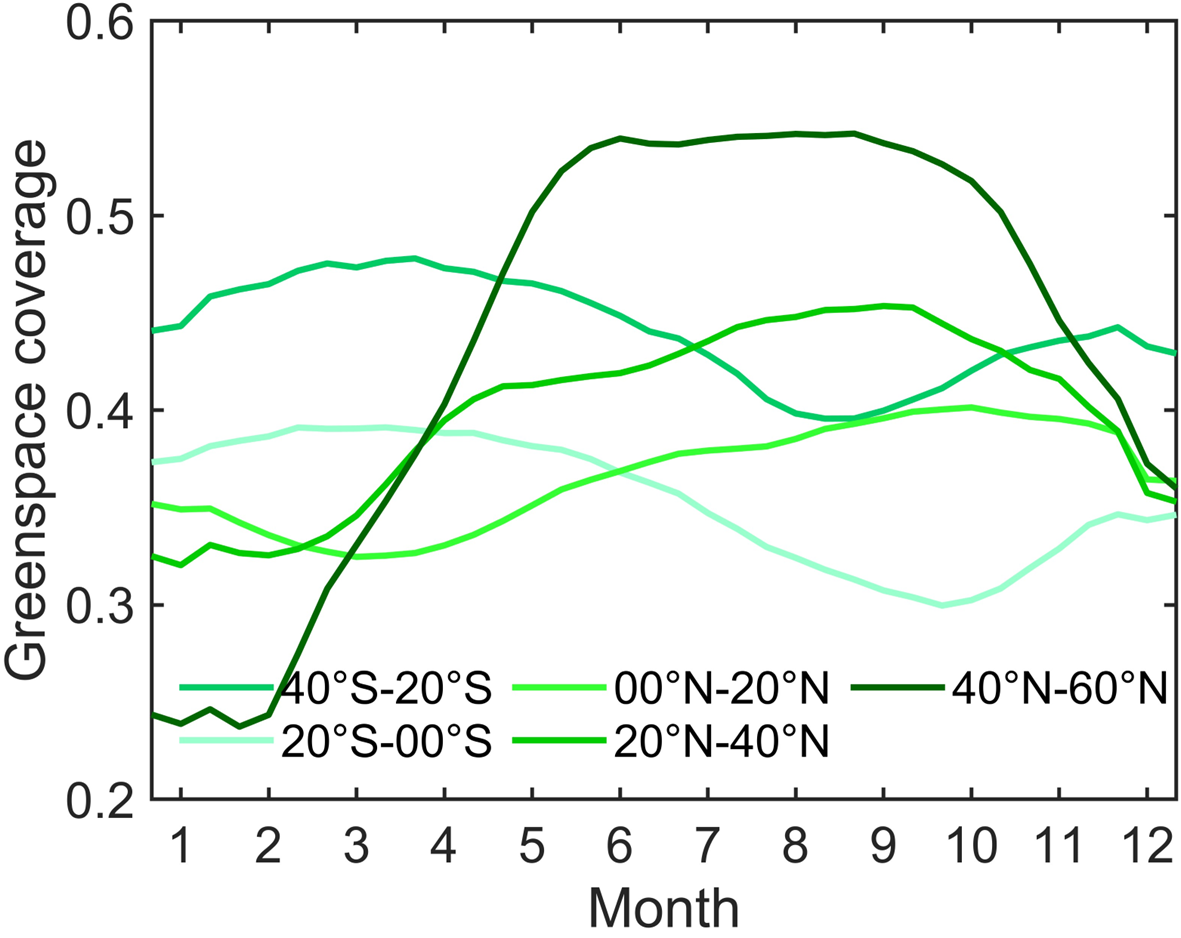


**Fig. S5.** Seasonal dynamic of city-level mean greenspace in 2021 across 5 climate zones, including temperate (40°N-60°N), subtropical (-40°S-20°S and 20°N-40°N), and tropical (-20°S-0°S and 0°N-20°N).


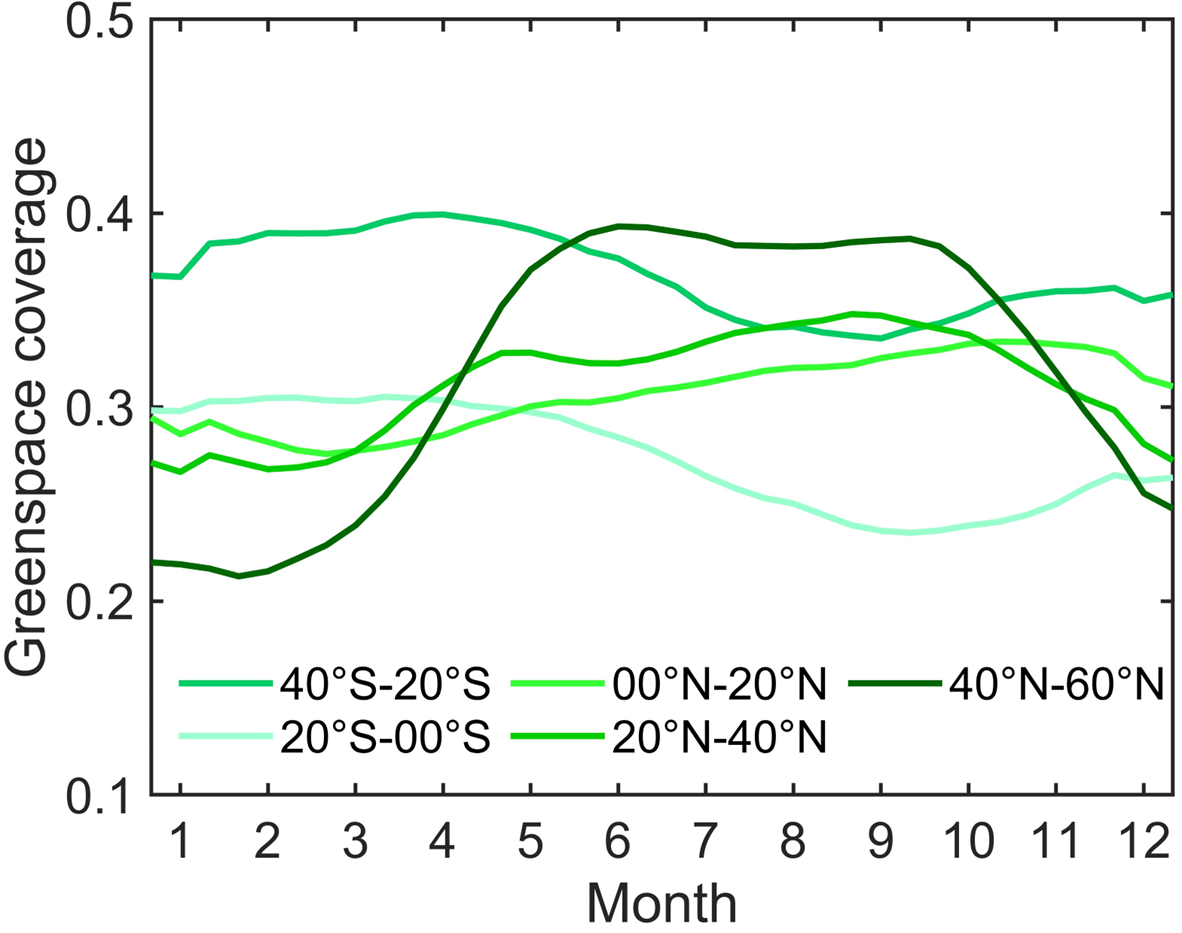


**Fig. S6.** Seasonal dynamic of city-level mean greenspace in 2022 across 5 climate zones, including temperate (40°N-60°N), subtropical (-40°S-20°S and 20°N-40°N), and tropical (-20°S-0°S and 0°N-20°N).


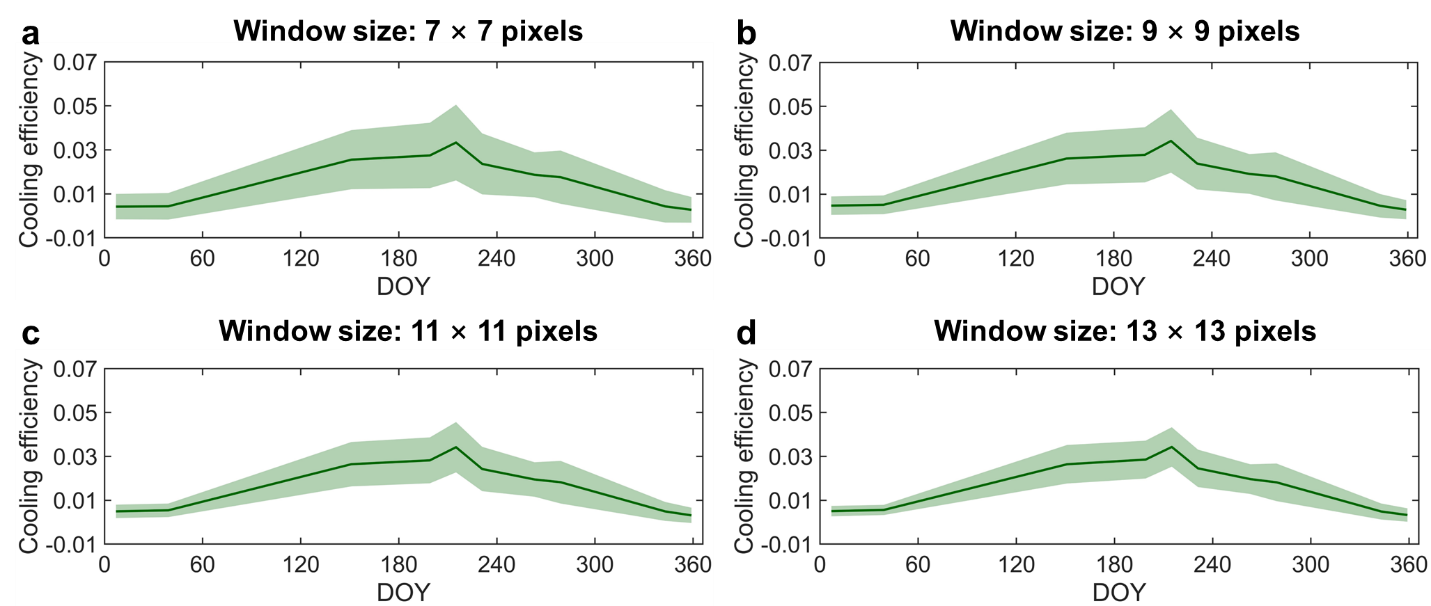


**Fig. S7.** Sensitivity analysis on the seasonality of greenspace cooling efficiency (CE), with different moving window sizes of **(a)** 7×7, **(b)** 9×9, **(c)** 11×11, and **(d)** 13×13 pixels.
